# Ensemble Improved Permutation Entropy: A New Approach for Time Series Analysis

**DOI:** 10.3390/e25081175

**Published:** 2023-08-07

**Authors:** Zhe Chen, Xiaodong Ma, Jielin Fu, Yaan Li

**Affiliations:** 1 School of Information and Communication, Guilin University of Electronic Technology, Guilin 541004, China; mxd1090163110@163.com (X.M.);; 2 Key Lab. of Cognitive Radio & Information Processing, The Ministry of Education, Guilin University of Electronic Technology, Guilin 541004, China; 3 School of Marine Science and Technology, Northwestern Polytechnical University, Xi’an 710072, China; liyaan@nwpu.edu.cn

**Keywords:** ensemble improved permutation entropy, feature extraction, data analysis

## Abstract

Entropy quantification approaches have gained considerable attention in engineering applications. However, certain limitations persist, including the strong dependence on parameter selection, limited discriminating power, and low robustness to noise. To alleviate these issues, this paper introduces two novel algorithms for time series analysis: the ensemble improved permutation entropy (EIPE) and multiscale EIPE (MEIPE). Our approaches employ a new symbolization process that considers both permutation relations and amplitude information. Additionally, the ensemble technique is utilized to reduce the dependence on parameter selection. We performed a comprehensive evaluation of the proposed methods using various synthetic and experimental signals. The results illustrate that EIPE is capable of distinguishing white, pink, and brown noise with a smaller number of samples compared to traditional entropy algorithms. Furthermore, EIPE displays the potential to discriminate between regular and non-regular dynamics. Notably, when compared to permutation entropy, weighted permutation entropy, and dispersion entropy, EIPE exhibits superior robustness against noise. In practical applications, such as RR interval data classification, bearing fault diagnosis, marine vessel identification, and electroencephalographic (EEG) signal classification, the proposed methods demonstrate better discriminating power compared to conventional entropy measures. These promising findings validate the effectiveness and potential of the algorithms proposed in this paper.

## 1. Introduction

It is widely recognized that analyzing time series generated from complex systems is an effective way to gain insight into underlying dynamics [1,2]. Numerous methods have been proposed for this purpose, including power spectrum analysis, short-time Fourier transform, wavelet transform, Lyapunov exponents [3], fractal dimensions [4], and entropy techniques [5,6,7,8,9,10,11,12,13,14,15,16,17]. Among them, entropy techniques have garnered increasing attention due to their ability to evaluate irregularity (or complexity) within time series and their potential for system identification.

Typically, entropy quantification approaches are developed through the following steps: (I) finding events from the data; (II) computing the probability distribution of such events; and (III) mapping the probability distribution to a single value. Conditional entropy and Shannon entropy are the most widely used entropy definitions for such a mapping, while the former provides the rate of information production, and the latter measures the amount of information. Various entropy algorithms have been proposed in the past years based on the two definitions mentioned above, such as approximate entropy (ApEn) [8], sample entropy (SampEn) [9], fuzzy entropy (FuzEn) [10], quadratic sample entropy [11], distribution entropy [12], permutation entropy (PE) [13], weighted permutation entropy (WPE) [14], modified permutation entropy [15], dispersion entropy (DispEn) [16], and fluctuation dispersion entropy (FDispEn) [17]. The development of new entropy metrics has shed new light on a wide range of engineering problems, including fault diagnosis [18,19], underwater target recognition [1,7,20,21,22], stock market analysis [23,24], and biomedical signal processing [8,9,15,25], among others.

Despite the great success that entropy algorithms have achieved in practical applications, they continue to face certain limitations that require further refinement. For instance, both ApEn and SampEn are sensitive to tolerance r, a parameter that decides the level of similarity between two vectors in the phase space [1,5,10]. If the value of tolerance is set too low, very few vectors are regarded as similar, leading to unreliable or undefined conditional entropy estimates. The situation can be worse if the data length is short. By contrast, a larger value of tolerance may result in a loss of information. FuzEn has been proposed as a solution to this issue by using the exponential function instead of the Heaviside function to obtain a fuzzy measurement of two vectors’ similarity [10]. However, FuzEn still requires pairwise similarity checks between vectors in the phase space; its computation cost increases quadratically with data length. An alternative approach, PE [13], uses the Bandt–Pompe procedure to symbolize the vectors based on the order of amplitudes, resulting in ordinal patterns (or permutation patterns). Despite its simplicity and computational efficiency, absolute amplitude information is overlooked in this process [14,26]. Some researchers also claimed that PE is liable to be affected by noise because the permutation relations can be varied by a small change in amplitude values [5,16]. Additionally, there are studies that have proved that PE is susceptible to the equal values in time series [15,27,28]. Typically, ranking the equal values according to their temporal order or breaking them by adding random perturbations are common ways to circumvent this problem [1,13]. Unluckily, a recent study pointed out that these solutions can lead to misinterpretations of the underlying nature of the electroencephalogram records [28]. Many efforts have been made by researchers to tackle the above-mentioned defects of PE. Fadlallah et al. proposed WPE [14], in which the amplitude information is considered by weighting the ordinal patterns. Bian et al. invented mPE [15], where the same symbols are assigned to the ties. Because of that, mPE can provide more potential motifs to represent the sub-series, and its ability to recognize the heart rate variability (HRV) signal is thus improved. Notably, both WPE and mPE have been shown to be insufficient in completely addressing the limitations inherent to PE, highlighting the need for further research and development in this area. Recently, DispEn [16] and its extension FDispEn [17] were devised by Hamed Azami and the coauthors, whose main idea is to represent the univariate time series with a small set of symbols. Then, entropy estimation of the original data can be equivalent to studying the probability distribution of symbol sequences and calculating the corresponding entropy value. Since the data are transformed into a new time series based on symbolic dynamics, some detailed information might be lost. Moreover, how to determine the number of symbols remains a problem. Therefore, each entropy approach has its advantages and limitations.

To enhance the performance of traditional entropy algorithms, a novel entropy measure called ensemble improved permutation entropy (EIPE) is proposed in this paper. We start by presenting a new data symbolization method that uses a symbol set composed of L elements to represent vectors in the phase space, resulting in symbolic patterns. It is imperative to note that the obtained symbolic patterns take both permutation relation and amplitude information into account. Then, like what is performed in the PE algorithm, the probability distribution of the symbolic patterns is mapped to an entropy value based on the Shannon entropy. However, one needs to artificially pre-define the discretization factor in the symbolization process, and determining this parameter remains a challenge. We addressed this issue by drawing inspiration from the ensemble technique presented in reference [5], where we varied the discretization factor and averaged the corresponding entropy results, resulting in the EIPE. To facilitate the analysis of signals over multiple temporal scales, a multiscale EIPE (MEIPE) algorithm is further introduced, where the coarse-graining technique is applied prior to the EIPE calculation. The effectiveness of the proposed methods is evaluated using various synthetic and experimental data, including RR interval data, bearing fault signals, underwater acoustic signals, and EEG signals.

The remainder of this paper is organized as follows: the proposed EIPE and MEIPE algorithms are described in Section 2; simulation and experimental results are provided in Section 3 and Section 4, respectively; and the paper is concluded in Section 5.

## 2. Methodology

### 2.1. Ensemble Improved Permutation Entropy

The EIPE algorithm is calculated through the following steps:

**Step 1.** As shown in Equation (1), given a univariate time series $x=\{x_{1},x_{2},\cdots ,x_{N}\}$, the cumulative distribution function is utilized for data normalization:

(1) $$y_{i}=\frac{1}{\sigma \sqrt{2\pi }}\int_{-\infty }^{x_{i}}e^{\frac{-{\left(t-\mu \right)}^{2}}{2\sigma^{2}}}dt,$$

where $y_{i}$ represents ith element of the normalized sequence y and μ and $\sigma^{2}$ denote the mean and variance of x, respectively.

**Step 2.** With embedding dimension m and time delay τ given, the reconstructed phase space is denoted by

(2) $$Y(j,:)=[y_{j},y_{j+\tau },\cdots y_{j+(m-1)\tau }]$$

where Y(j,:) is the jth row of Y, and j=1,2,⋯N−(m−1)τ.

**Step 3.** Let $y_{\max}$ and $y_{\min}$ represent the maximum and minimum values of y, respectively, and L be the discretization factor (an artificially pre-defined parameter); the uniform partition function (UPF) is defined as follows:

(3) $$UPF(u)=\{\begin{array}{cc} 0 & y_{\min}\leq u<\Delta +y_{\min}\\ 1 & y_{\min}+\Delta \leq u<2\Delta +y_{\min}\\ \vdots & \vdots \\ L-1 & y_{\max}-\Delta <u\leq y_{\max} \end{array}$$

where $\Delta =(y_{\max}-y_{\min})/L$. Obviously, for arbitrary input $u\in (y_{\min},y_{\max})$, UPF converts it into an integer symbol ranging from 0 to L−1. Let the first column of Y be the input of UPF; Y(:,1) is then transformed into a symbol sequence, represented as S(:,1).

**Step 4.** For the kth column of Y, indicated as Y(:,k), 2≤k≤m, its corresponding symbolization result S(:,k) is achieved by Equation (4):

(4) S(j,k)=S(j,1)+⌊(Y(j,k)−Y(j,1))/Δ⌋

where 1≤j≤N−(m−1)τ, and ⌊⌋ represents a function that rounds the elements in it to the nearest integers towards zero. Upon completion of the symbolization process for all components within the phase space Y, the resulting entity, expressed as the symbolic phase space S, is obtained. Further, each row of S is referred to as a symbolic pattern (SP), which incorporates both permutation relation and amplitude information.

**Step 5.** As shown in Equation (5), the probability distribution of SP is computed and then mapped to an entropy value based on the definition of Shannon entropy. This resulting entropy value is referred to as the improved permutation entropy (IPE). Since each symbolic pattern comprises m elements, and each element can take L possible states, the total number of symbolic patterns is thus given by $L^{m}$. It is apparent that the IPE attains its maximum value only when SP follows a uniform distribution. To optimize the IPE, a normalization technique can be applied using Equation (6).

(5) $$IPE(m,L,\tau )=-\sum_{l=1}^{L^{m}}p_{l}\ln(p_{l})$$

(6) $$IPE(m,L,\tau )=-\sum_{l=1}^{L^{m}}p_{l}\ln(p_{l})/\ln(L^{m})$$

The above description indicates that the discretization factor L has a significant impact on the calculation of the IPE, because it plays a pivotal role in the symbolization process, as depicted in Equations (3) and (4). Evidently, a higher value of L leads to a comparatively lesser loss of time series’ information during the symbolization process, while a smaller L value offers better noise resistance, albeit at the cost of losing some information. The selection of an appropriate discretization factor L depends on the characteristics of the signal, including its signal-to-noise ratio (SNR). Unfortunately, this a priori information is usually unknown. The ensemble technique, which involves the integration of multiple methods to improve overall prediction performance, can address this issue. Motivated by this idea, we propose the EIPE. As can be seen in Equation (7), EIPE is calculated as the mean of the IPE results derived from varying values of L.

(7) $$EIPE(m,\tau )=\frac{1}{b-a+1}\sum_{i=a}^{b}IPE(m,i,\tau )$$

where a and b are the minimum and maximum value of L, respectively.

### 2.2. Multiscale Ensemble Improved Permutation Entropy

Complex time series often have intricate structures across multiple temporal scales, which conventional entropy measures that rely on a single-scale analysis fail to account for. To remedy this, multiscale ensemble improved permutation entropy (MEIPE) is proposed in this section, where a coarse-graining process [25] is conducted prior to a comprehensive analysis with EIPE. The coarse-graining process of a time series $x=\{x_{1},x_{2},\cdots ,x_{N}\}$ is given by Equation (8), where $r_{}^{s}$ represents the output sequence under scale s. Applying EIPE to process the subsequence $r_{}^{s}$, the obtained result $EIPE_{}^{s}$ is the entropy of the original sequence under scale s. This process is repeated for all scale factors, resulting in an entropy vector, namely the MEIPE. In other words, MEIPE is essentially a plot of EIPE versus scale factors.

(8) $$r_{i}^{s}=\frac{1}{s}\sum_{i=(j-1)s+1}^{js}x_{i}$$

## 3. Synthetic Data Analysis

In this section, the effectiveness of the proposed EIPE algorithm is verified through several synthetic signals. As can be seen in Equation (6), embedding dimension, time lag, and discretization factor need to be properly set to implement the EIPE algorithm. According to the conclusions in [1,5,13], 3≤m≤7 and τ=1 are recommended. In what follows, unless otherwise specified, we varied the discretization factor L from 2 to 8 and set m=4 and τ=1.

### 3.1. Noise Signals

Noise is ubiquitous in various systems and applications. White, pink, and brown noise are the most frequently used random signals for model analysis [5,29]. White noise is a type of noise that contains equal energy or power across all frequencies; its power spectrum density can be represented as $S_{w}(f)=C_{w}$, where $C_{w}$ is a constant. Pink noise, also known as 1/f noise, is a type of noise where the power of the signal decreases by 3 decibels per octave as the frequency increases. Compared with pink noise, brown noise has a lower intensity at higher frequencies. The power spectrum density of pink and brown noise can be denoted by $S_{p}(f)=C_{p}/f$ and $S_{b}(f)=C_{b}/f^{2}$, respectively, where $C_{p}$ and $C_{b}$ are constants.

**Figure 1:**
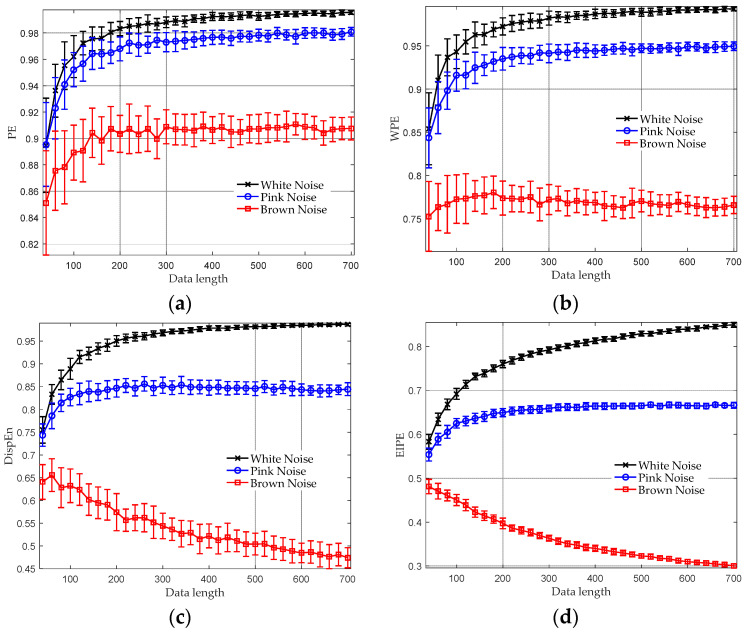
Comparative results of diverse entropy algorithms regarding their discriminative capability among white, pink, and brown noise. (**a**) PE analysis result; (**b**) WPE analysis result; (**c**) DispEn analysis result; and (**d**) EIPE analysis result.

The comparative results of diverse entropy algorithms in terms of their ability to discriminate between three types of noise are presented in Figure 1. The average entropy values, along with their error bars representing the standard deviation (SD), are plotted against the varying data length. The data length was changed from 40 to 700, with an increment of 20. For each data length, 40 independent realizations were generated for each type of noise. As can be seen, no matter which algorithm is utilized, white noise attains the highest entropy values, followed by pink and brown noise. This result is consistent with the reality that white noise is the most complex, succeeded by pink and brown noise [5,29]. It can also be observed that EIPE requires fewer samples than the other methods to discriminate between the three types of noise, implying that our method has a low dependency on data length and can extract effective features of the noises even with limited samples.

### 3.2. Logistic Map

The logistic map can be described as $x_{n+1}=\mu x_{n}(1-x_{n})$, where μ is a parameter that controls the dynamic behavior of the model. According to previous studies [30,31,32], when μ increases from 3.5 to 3.99, the model exhibits a period-doubling bifurcation. In particular, for 3.57≤μ≤3.99, the system is chaotic, except for rare exceptions like μ≈3.84.

**Figure 2:**
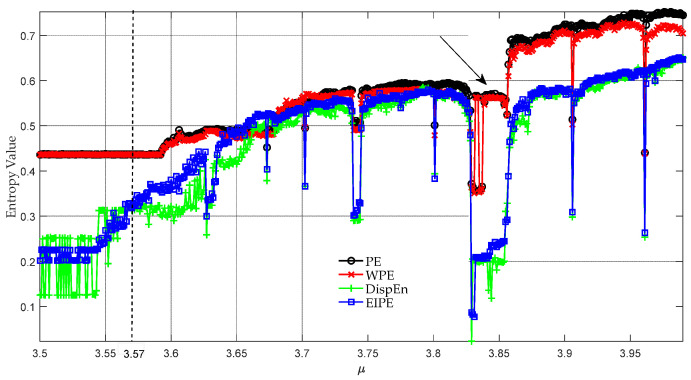
Plot of entropy versus μ for the logistic map with 3.5≤μ≤3.99. The arrow indicates the specific region where the behavior of the system transitions from chaotic to periodic.

To evaluate the ability of the EIPE algorithm to detect periodicity and nonlinearity, we varied μ from 3.5 to 3.99 with a step size of Δμ=0.001. For each μ, we generated a time series with 10,000 sampling points and computed its entropy. Figure 2 shows how the entropy values obtained by different algorithms change with μ. When μ≈3.57, the EIPE exhibits a positive correlation with the augmentation of μ, affirming that the system progressively grows in complexity. This phenomenon agrees with the fact that the system undergoes a transition from periodic to chaotic behavior [32]. Remarkably, the values of the other three entropy algorithms remain unaltered in this context. When μ≈3.84, both DispEn and EIPE exhibit a significant decline in this region, whereas PE and WPE initially decrease but quickly rebound afterward. It is noteworthy to mention that the profile obtained by the EIPE algorithm is consistent with the result depicted in Figure 1 of reference [32], signifying the potential of the proposed method in discriminating between regular and non-regular dynamics.

### 3.3. Noisy Lorenz Signal

**Figure 3:**
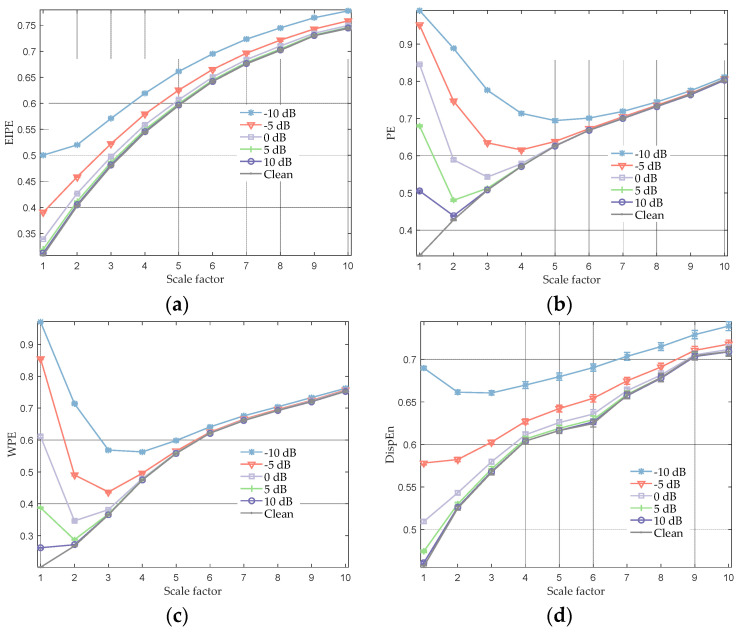
Multiscale entropy analysis of Lorenz time series under different SNR conditions. (**a**) MEIPE analysis result; (**b**) multiscale PE analysis result; (**c**) multiscale WPE analysis result; and (**d**) multiscale DispEn analysis result.

To evaluate the performance of the proposed algorithm under noisy conditions, we added white Gaussian noise into the Lorenz time series to generate signals at different SNR levels. A fourth-order Runge–Kutta scheme with a time step of Δt=0.001 was applied to solve the Lorenz system depicted in Equation (9), and 50,000 data points were recorded. For each SNR condition, 40 trials were independently conducted, and their multiscale entropies were calculated through various approaches. The average multiscale entropy values with their SD error bars are demonstrated in Figure 3. For all entropy algorithms, the multiscale entropy curve increases as the SNR decreases. Notably, from the results depicted in Figure 3a, it Is evident that the MEIPE curve at −10 dB remains close to that of the clean signal, suggesting the minimal influence of noise on the performance of the MEIPE algorithm. Conversely, the other three approaches display larger deviations in entropy values under low SNR conditions, especially for lower-scale factors. The findings in Figure 3 illustrate the robustness of the MEIPE algorithm against the noise.

(9) $$\{\begin{array}{c} \dot{x}=10\left(y-x\right)\\ \dot{y}=x\left(28-z\right)-y\\ \dot{z}=xy-\frac{8}{3}z \end{array}$$

## 4. Experimental Data Analysis

In this section, the proposed EIPE and MEIPE algorithms are applied to process three kinds of experimental data: RR intervals, bearing fault signals, underwater acoustic signals, and EEG signals. All these data are regarded as complex time series.

### 4.1. RR Intervals

**Figure 4:**
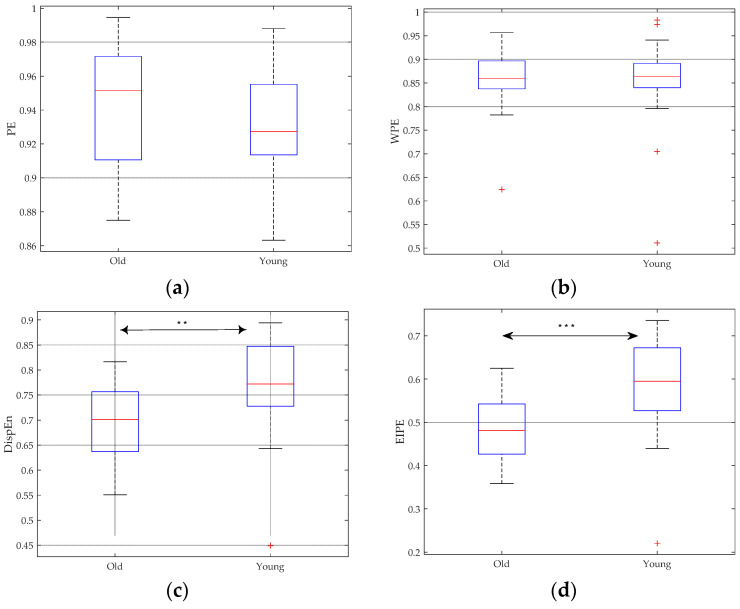
Boxplots of distinct entropy approaches computed from the RR intervals of healthy young and healthy elderly participants. (**a**) PE analysis result; (**b**) WPE analysis result; (**c**) DispEn analysis result; and (**d**) EIPE analysis result. *p*-values smaller than 0.01 and 0.001 are represented by ** and ***, respectively.

The RR interval data used in this paper originate from the Fantasia dataset [33]. This collection comprises RR interval data from 20 young and 20 elderly healthy participants, with their ages ranging from 21 to 34 and 68 to 85, respectively. Both the DispEn and EIPE analysis results, as shown in Figure 4c and d, respectively, illustrate that the RR intervals of healthy young subjects exhibit greater irregularity in comparison to those of healthy elderly individuals. However, the PE and WPE analysis results show insignificant differences between the two groups.

To quantitatively assess the differences between entropy values for young and elderly individuals, the non-parametric Mann–Whitney U-test is utilized. The significance of inter-group differences can be determined through the *p*-values, with lower *p*-values indicating more significant distinctions. In Figure 4, *p*-values smaller than 0.01 and 0.001 are represented by ** and ***, respectively. The calculated *p*-values corroborate the visual observations from the boxplots, where the *p*-values for PE and WPE are greater than 0.05 (0.2792 and 0.8498). On the other hand, DispEn and EIPE yield *p*-values of 0.0038 and 0.000437, respectively, providing strong evidence for their exceptional discriminability in distinguishing between the two types of signals.

### 4.2. Bearing Fault Signals

In this subsection, a collection of bearing fault signals originating from the Case Western Reserve University Bearing Data Center is analyzed. The collection contains four categories of signals that are normal, ball fault (BF), inner race fault (IRF), and outer race fault (ORF) [34]. The motor speed is about 1730 r/min, and the fault diameter is 0.1778 mm.

**Figure 5:**
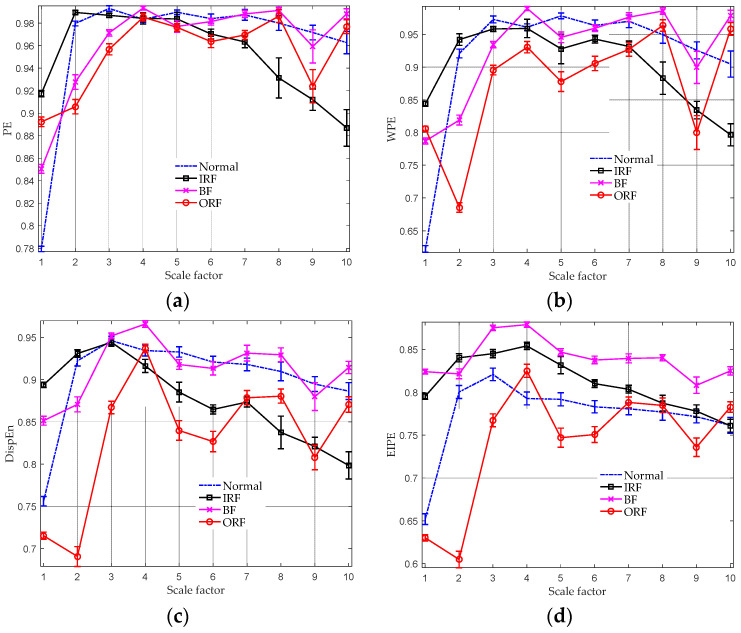
Multiscale entropy analysis results of four types of bearing fault signals. (**a**) Multiscale PE analysis result; (**b**) multiscale WPE analysis result; (**c**) multiscale DispEn analysis result; and (**d**) MEIPE analysis result.

Each type of signal consists of approximately 120,000 data points. To facilitate analysis, each datum was divided into 10 equally sized segments, with each segment containing 12,000 sample points. As can be seen in Figure 5d, the EIPE values of BF remain relatively constant across all scales. In contrast, the EIPE values of IRF increase slightly between scales 1 and 4 and then decrease persistently. For the normal category, the EIPE values increase sharply (from 0.65 to 0.8) at lower scales and then show a minor decline. The MEIPE feature of ORF signals shows a decrease initially, followed by oscillations between scales 2 and 10. The distinct underlying structures of different bearing fault signals make their MEIPE curves unique, both in terms of the entropy magnitude and the variation trend across the scale factors. For comparison, analysis results of other multiscale entropy approaches are also provided in Figure 5. Obviously, the entropy curve seems closer to each other in the multiscale PE and multiscale WPE results. For instance, at scale 7, these algorithms assign high entropy values (≈0.98) to normal and BF signals, making them indistinguishable. Although multiscale DispEn outperforms multiscale PE and multiscale WPE, its separability declines under the scales 2, 3, 4, 7, 9, and 10, where entropy features of distinct types of signals overlap with each other. By contrast, the proposed MEIPE algorithm can distinguish four types of signals at most scales. This finding suggests that the MEIPE algorithm has the potential for bearing fault diagnosis.

### 4.3. Underwater Acoustic Signals

Identifying targets based on their emitted sound poses a significant challenge in underwater acoustic signal processing [1,4,7], primarily due to the complex ocean environment and the presence of high ambient noise levels. In this subsection, we adopted the MEIPE algorithm to analyze three types of ship-generated noise, namely, from passenger ships, ocean liners, and motorboats [35]. For the sake of simplicity, the dataset was divided into various segments, with each segment lasting for 3 s. Given a sampling frequency of 52,734 Hz, each segment consisted of 158,202 sample points. Additional details regarding the dataset can be found in Table 1. Notably, signals from various distinct marine vessels were collected for each category.

**Figure 6:**
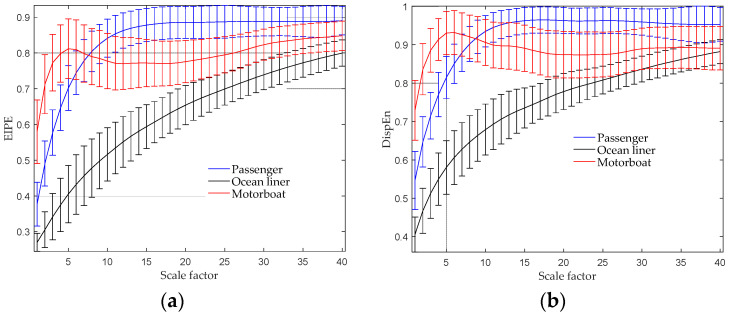
Multiscale entropy analysis results of three types of ship-radiated noise. (**a**) MEIPE analysis result; (**b**) multiscale DispEn analysis result.

The MEIPE analysis result is presented in Figure 6a, where the scale factor is set to 40. The plot displays the average EIPE values versus the scale factor, accompanied by their corresponding SD error bars. The EIPE value of the ocean liner increases consistently across all scale factors. On the other hand, the EIPE value of the passenger ship shows a sharp increase and then remains relatively constant after scale 15. Interestingly, the EIPE value of the motorboat exhibits an initial increase from scales 1 to 5, followed by a downward spike from scales 5 to 35. Visually examining the MEIPE curves, it can be observed that the curves for the three target categories are distinct from each other, indicating the excellent discriminating power of our proposed method. For comparison, the multiscale DispEn analysis result is presented in Figure 6b, which shows similar trends as the MEIPE analysis result. However, there are some subtle differences observed between scales 16 and 25, where the multiscale DispEn features of three of the ships are closer to each other when compared to the MEIPE features.

To further quantify the discriminative capability of the MEIPE features for the three categories of ships, we employed a probabilistic neural network (PNN) for feature training and recognition. For testing, 150 randomly selected segments were retained for each target category, while the remaining segments were used for network training. The recognition results of the network are presented in Table 2. For comparison, the classification results of the multiscale DispEn algorithm are given in Table 3.

The results clearly indicate that both the MEIPE and multiscale DispEn algorithms achieve an impressive recognition rate of 100% for the passenger ship category. However, for the motorboat and ocean liner categories, the multiscale DispEn algorithm demonstrates a comparatively lower recognition rate, denoted as 74% and 82%, respectively, in contrast to the MEIPE algorithm. Overall, the MEIPE algorithm attains a classification accuracy of 92.44% for the three target categories, which is 7.11% higher than multiscale DispEn. These findings illustrate the superior performance of the MEIPE algorithm in accurately identifying and discriminating between the various ship categories. 

### 4.4. EEG Signals

EEG records contain fruitful physiological and pathological information. The analysis of EEG signals is of high significance in numerous applications, such as evaluating the mental state of subjects, assessing drivers’ fatigue, measuring anesthesia depth, and predicting the onset of epileptic seizures [6]. In this subsection, our proposed algorithm was employed to process the commonly used University of Bonn EEG database. Our analysis covered four subsets of the database, which correspond to healthy subjects with eyes open (Class 0), healthy participants with eyes closed (Class 1), subjects during interictal epileptic activity (Class 2), and participants experiencing seizure attacks (Class 3), respectively. Each subset comprises 100 data segments, with each segment lasting 23.6 seconds and consisting of 4097 data points (sampling frequency is 173.61 Hz). For detailed descriptions of the dataset, please see reference [6].

**Figure 7:**
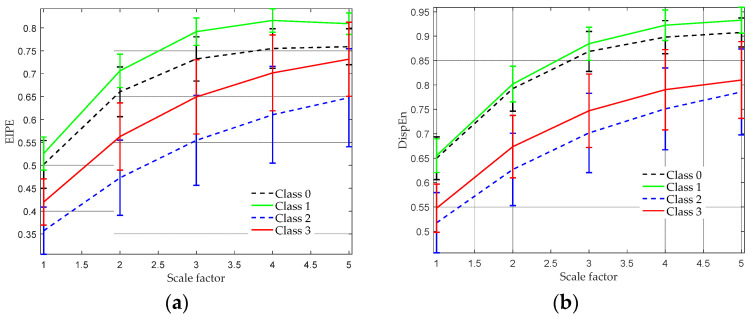
Multiscale entropy analysis results of four types of EEG signals. (**a**) MEIPE analysis result; (**b**) multiscale DispEn analysis result.

The MEIPE analysis result is presented in Figure 7a, where the scale factor is varied from 1 to 5, owing to the limited length of the signal. It is evident that Class 1 acquires the highest EIPE values across all scale factors, followed by Class 0, 3, and 2. Notably, the MEIPE features of each category exhibit a distinct separation from one another. In contrast, Figure 7b reveals that the multiscale DispEn features of Class 0 and 1 are challenging to discriminate, particularly for scales 1 to 3. Additionally, under scales 3 to 5, the differences between Class 2 and 3 appear less pronounced. These outcomes indicate that the proposed MEIPE algorithm may be better suited for discriminating between different EEG classes in comparison to multiscale DispEn.

To quantitatively evaluate the differences in entropy values across different EEG categories, the non-parametric Mann–Whitney U-test is utilized, and the corresponding *p*-values are listed in Table 4 and Table 5. These statistical results are in line with the findings in Figure 7. With the application of multiscale DispEn, it is observed that there are no significant differences between Class 2 and 3 at scales 1 and 2. Furthermore, the distinction between Class 0 and 1 at scale 5 is not pronounced. In contrast, the inter-group differences are found to be significant across all scale factors when using the MEIPE approach. Based on these findings, we can confidently conclude that MEIPE outperforms multiscale DispEn in accurately discriminating between EEG categories.

## 5. Conclusions

To enhance the performance of traditional entropy quantification methods, this paper introduces two novel algorithms for time series analysis: the EIPE and MEIPE. To validate the effectiveness of these proposed methods, a comprehensive evaluation was conducted using both simulated and experimental signals. The findings of this study demonstrate that the EIPE algorithm outperforms traditional entropy algorithms in distinguishing white, pink, and brown noise, even with a smaller number of samples. Moreover, EIPE exhibits sensitivity to the underlying behavior of the model, making it effective in discriminating between regular and non-regular dynamics. Additionally, the MEIPE algorithm exhibits reduced dependence on SNR levels, enabling its application in noisy conditions. Finally, the proposed methods demonstrate better discriminating power compared to conventional entropy measures in practical applications, such as RR interval data classification, bearing fault diagnosis, marine vessel identification, and EEG signal classification. Hence, the EIPE and MEIPE algorithms are of value in time series analysis.

## Figures and Tables

**Table 1:** Description of three types of ship-radiated noise.

Categories	Ship Name	Number of Segments
Passenger	Mar de Cangas	267
	Mar de Onza	124
	Pirata de Salvora	65
	Arrois	103
Ocean liner	MSC Opera	160
	Adventure of the sea	89
	Costa Voyager	397
Motorboat	Small Yacht	76
	Motorboat2	86
	High-speed motorboat	36
	Zodiac	96

**Table 2:** PNN classification results for three types of ships using MEIPE features.

Categories	Recognized as			Classification Accuracy
	Passenger	Ocean Liner	Motorboat	
Passenger	150	0	0	100%
Ocean liner	23	127	0	84.67%
Motorboat	6	5	139	92.67%
In total	-	-	-	92.44%

**Table 3:** PNN classification results for three types of ships using multiscale DispEn features.

Categories	Recognized as			Classification Accuracy
	Passenger	Ocean Liner	Motorboat	
Passenger	150	0	0	100%
Ocean liner	27	123	0	82%
Motorboat	30	9	111	74%
In total	-	-	-	85.33%

**Table 4:** The significance of inter-group differences between distinct categories when MEIPE is applied. *p*-values smaller than 0.001 are represented by ***.

	Scale 1	Scale 2	Scale 3	Scale 4	Scale 5
Class 0 vs. Class 1	*p* = 5.4 × 10^−14^ ***	*p* = 1.5 × 10^−12^ ***	*p* = 1.9 × 10^−11^ ***	*p* = 4.8 × 10^−11^ ***	*p* = 1.5 × 10^−10^ ***
Class 2 vs. Class 3	*p* = 2.5 × 10^−5^ ***	*p* = 1.2 × 10^−10^ ***	*p* = 2.3 × 10^−18^ ***	*p* = 5.6 × 10^−24^ ***	*p* = 6.4 × 10^−21^ ***

**Table 5:** The significance of inter-group differences between distinct categories when multiscale DispEn is applied. *p*-values smaller than 0.01, and 0.001 are represented by ** and ***, respectively.

	Scale 1	Scale 2	Scale 3	Scale 4	Scale 5
Class 0 vs. Class 1	*p* = 0.0025 **	*p* = 6.4 × 10^−5^ ***	*p* = 8.3 × 10^−4^ ***	*p* = 0.0081 **	***p* = 0.1314**
Class 2 vs. Class 3	***p* = 0.1582**	***p* = 0.0739**	*p* = 0.0043 **	*p* = 6.2 × 10^−7^ ***	*p* = 4.3 × 10^−9^ ***

## Data Availability

The data used to support the findings of this study are available from the corresponding author upon request.
